# Improved Artificial Bee Colony Algorithm Based Gravity Matching Navigation Method

**DOI:** 10.3390/s140712968

**Published:** 2014-07-18

**Authors:** Wei Gao, Bo Zhao, Guang Tao Zhou, Qiu Ying Wang, Chun Yang Yu

**Affiliations:** College of Automation, Harbin Engineering University, Harbin 150001, China; E-Mails: gaowei@hrbeu.edu.cn (W.G.); zhaobo880928@126.com (B.Z.); wqy869087@163.com (Q.Y.W.); chunyang.yu@hotmail.com (C.Y.Y.)

**Keywords:** gravity matching navigation, ABC, gravity anomaly, local single point search, overall restriction

## Abstract

Gravity matching navigation algorithm is one of the key technologies for gravity aided inertial navigation systems. With the development of intelligent algorithms, the powerful search ability of the Artificial Bee Colony (ABC) algorithm makes it possible to be applied to the gravity matching navigation field. However, existing search mechanisms of basic ABC algorithms cannot meet the need for high accuracy in gravity aided navigation. Firstly, proper modifications are proposed to improve the performance of the basic ABC algorithm. Secondly, a new search mechanism is presented in this paper which is based on an improved ABC algorithm using external speed information. At last, modified Hausdorff distance is introduced to screen the possible matching results. Both simulations and ocean experiments verify the feasibility of the method, and results show that the matching rate of the method is high enough to obtain a precise matching position.

## 1. Introduction

Gravity matching navigation is a kind of matching navigation technology aimed at obtaining the best matching position, in which the gravity data from the gravimeter and gravity reference database are imported into the computer for gravity matching solver. Gravity matching navigation has always been a key research area for underwater passive navigation, and in general, methods of gravity matching include recursive filtering method and correlation matching method. For the recursive filtering method, the precise model of gravity anomaly is difficult to establish, and its application is limited. Meanwhile, for the correlation matching method, the computational complexity is high, and the matching accuracy is low [1]. With the development of intelligent algorithms, many scholars are keen to do research on their application in the field of navigation. Particle swarm algorithm is applied to the path planning, solving local optimum problem and decreasing the high complexity of existing algorithms [2]. A new star recognition method based on the Adaptive Ant Colony (AAC) algorithm has been developed to increase the star recognition speed and success rate for star sensors [3]. The Artificial Bee Colony (ABC) algorithm has been proposed for training RBF networks [4]. Multi-layer Feed-forward Neural Networks (MFNNs) is proposed in order to enhance the performance of low cost INS/GPS integrated systems [5]. A genetic algorithm is introduced into the selection of adaptation areas in geomagnetic aided navigation to achieve high recognition accuracy [6]. The problem of excessive search space in geomagnetic matching is solved by using the genetic algorithm to search the possible tracks [7]. Similar with geomagnetic matching navigation, gravity matching navigation uses the physical information of the Earth to achieve the matching position [8,9]. Therefore, the artificial intelligence algorithms are equal when applied to the search process of gravity matching with the advantages of the intelligent algorithms, in order to improve the performance of the gravity matching algorithm.

As one of the most recent intelligent algorithms, the ABC algorithm is able to replace the non intelligent search strategy of traditional gravity matching navigation to enhance the efficiency of gravity matching navigation for its fast convergence speed and low complexity. However, there are still some problems that need to be discussed which are similar to other intelligent algorithms (GA, EA, PSO); though the algorithm converges rapidly, it increases the possibility of falling into local optimum or prematurely due to evolution and selection strategy [10–13]. In recent years, many scholars have tried to improve the basic ABC algorithm. For example, the global best individual is used to guide the bees to increase the development ability of the ABC algorithm [14]. The initialization of bee colony with chaos and backward learning which is combined with differential evolution ideas is introduced to improve the diversity of the bee colony [15]. The inertia factor and acceleration factor are introduced to the ABC algorithm to increase the convergence rate [16]. Both the number of search parameters and the range of search steps are adjusted to improve search performance [17], in order to avoid the constraints of fixed-step search and increase the efficiency, especially for search objects with high-dimension parameters. These improvements are aimed at the search process for the entire bee colony, but for different search stages, the pressures of search and selection are different. This paper improves the search performance of the algorithm by adaptively adjusting the range of search steps and calculations of transition probability. Besides, to seek better performance of gravity matching based on the intelligent algorithm, modifications to the intelligent algorithm itself are not enough. What is more, the algorithm can yield the greatest returns through analysis of the application environment and reasonable design of the search mechanism. Considering the matching result is not exclusive, a new search mechanism is proposed in this paper combining multi-group local single point search with double-group overall constrained search. The experimental results show that the gravity matching method can solve the problem of mismatch, and achieve a precise matching position.

The paper is organized as follows. In Section 1, the basic principles of gravity matching are introduced, and the gravity matching with intelligent algorithms is presented. In Section 2, the basic ABC algorithm is described, and some modifications to the basic ABC algorithm are done to improve the search performance. In Section 3, the matching strategy based on improved ABC algorithm is introduced to make it more suitable for application to the matching algorithm. In Section 4, a simulation experiment and an ocean experiment are presented and the results are discussed, to verify the feasibility of the gravity matching algorithm.

## 2. Principle of Gravity Matching

**Figure 1.**
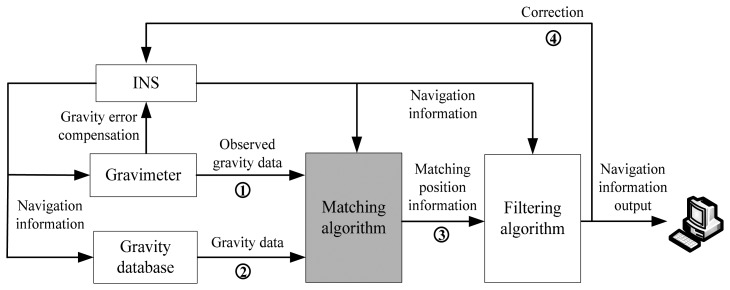
Schematic diagram of gravity matching.

Gravity aided navigation is an autonomous navigation technology using gravity feature information of the Earth to determine the position of the vehicle. As described in Figure 1, the key components of the gravity aided navigation system are inertial navigation system (INS), gravity reference database, gravity sensing device (gravimeter) and a computer for the matching calculation [18–20].

The basic working principles of gravity matching navigation include:

① Gravimeter measures the gravity anomaly data of the vehicle in the track;
② The corresponding gravity data is extracted from the gravity reference database stored in the computer according to the position information provided by INS;
③ Both types of gravity data are sent to the computer for matching calculation, and the computer will output the best matching position information;
④ This matching position information obtained is utilized to restrain the INS errors and enhance the navigation accuracy.

This paper mainly researches the matching algorithm in Figure 1, which is regarded as the core technology of gravity matching. In this work, intelligent search algorithm is applied to the search process matching points with full convergence speed, high search precision, good stability and other advantages.

## 3. Artificial Bee Colony Algorithm

### 3.1. Basic ABC Algorithm

The ABC algorithm, proposed by Karaboga from Turkey Erciyes University in 2005 for real-parameter optimization, is a recently introduced swarm intelligence algorithm. As the name implies, the ABC algorithm simulates the behaviors of the bee colony by observing how bees find the nectar, information exchange, and automatically assigning the number of groups of bees [14]. In Table 1, a one-to-one relationship exists between the behavior of bees and the optimization problem.

In the basic ABC algorithm, the minimal model in a bee colony that the algorithm simulates consists of three kinds of bees: employed bees, onlooker bees and scout bees. Employed bees are responsible for exploiting the nectar sources and giving information to onlooker bees in the hive about the quality of the food sources (a food source means a feasible solution for the problem with multiple solutions) they are exploiting. Onlooker bees choose a food source to exploit with a certain probability based on the information shared by the employed bees. Besides, Scouts randomly search the surrounding environment in order to find a better nectar source depending on an internal motivation or possible external clues.

At the initial stage of the search process, the bees start to explore the environment randomly in order to find a nectar source. After finding a nectar source, half of the bees become employed bees and prepare to exploit the discovered source. If the quality of the new position is better than the old, the new position will be memorized. If the source is exhausted, the bee becomes a scout and starts to randomly search for a new source. Onlooker bees choose the employed bees with a certain probability proportional to the quality of the source, and search for a new position around the neighborhood, and check the amount of honey. The bees try their best to find the best nectar with maximum amount of honey [14–17].

**Figure 2.**
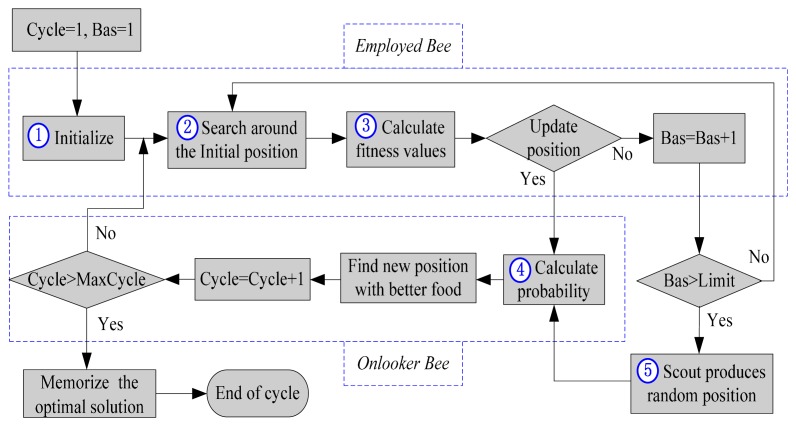
Flowchart of basic ABC algorithm.

All these units and interactions in the basic ABC algorithm are shown as a flowchart in Figure 2.

The key steps of the ABC algorithm in Figure 2 are described in detail. In Step ①, the algorithm starts with randomly produced nectar sources that correspond to the solutions in the search space, in which the parameters of the initial position are determined randomly within the range of the boundaries.

(1) $$x_{i}^{j}=x_{\text{INS}}^{j}+R^{j}\cdot \text{rand}(0,1)$$

where *i* = 1… *N*, *j* = 1…*D*, *N* is the number of nectar sources, *D* is the number of optimization parameters, $x_{\min}^{j}$ and $x_{\max}^{j}$ are the maximum and minimum of parameter *j*.

In Step ②, the employed bees and onlooker bees update their positions according to the following Equation:

(2) $$x_{i}^{j}=x_{i}^{j}+(x_{i}^{j}-x_{\text{neighbour}}^{j})\cdot rd$$

where *rd* is a uniformly distributed real random number in the range [−1, 1], *neighbour* ∈(1, *N*) is a randomly chosen, *j* is a random integer in the range [1, *D*].

If some parameter values which are produced by this operation exceed their predetermined boundaries, the parameter value is set to the corresponding boundary.

(3) $$x_{i}=\{\begin{array}{cc} x_{\min}^{i} & \;if\;x_{i}<x_{\min}^{i}\\ x_{\max}^{i} & \;if\;x_{i}>x_{\max}^{i} \end{array}$$

In Step ③, after a new position is produced within the boundaries, a fitness value will be assigned to the solution by Equation (4).

(4) $${\text{fit}}_{i}=\{\begin{array}{cc} 1/(1+f_{i}) & \;if\;f_{i}\geq 0\\ 1+\text{abs}(f_{i}) & if\;f_{i}<0 \end{array}$$

where *f_i_* is the cost value of the solution *i*, *fit_i_* is the fitness value of the solution *i* corresponding with the richness of the nectar source.

In Step ④, each employed bee is chosen by the onlooker bee with probability in size to the fitness value by Equation (5).

(5) $$P_{i}={\text{fit}}_{i}/\sum_{j=1}^{N}{\text{fit}}_{j}$$

In Step ➄, the best solution has not been updated when the search for matching point is assumed to be exhausted and will be replaced with a new nectar source discovered by the scout. This action is taken to increase the diversity and randomness of the bee colony. Specifically, the new nectar source is updated by Equation (6).

(6) $$x_{i}^{j}=x_{\text{INS}}+\text{rand}(0,1)R^{j}$$

### 3.2. Improved ABC Algorithm

In Figure 2, two main modifications to the basic ABC algorithm are done: adjusting the search strategy (number of search parameters and range of search step) and calculation of transition probability. Accordingly, adjusting the number of search parameters is mainly applied to the circumstance with high-dimensional parameters [21], however, only two parameters (longitude and latitude) in the gravity matching process are adjustable. Therefore, the basic ABC algorithm is improved in this paper by adjusting the range of the search step and calculation of transition probability.

#### 3.2.1. Adjusting the Range of Search Step *SF*

In the basic ABC algorithm, both employed bees and onlooker bees update their positions according to Equation (1), in which the range of the search step is a constant. It is understandable that the range of search step is too small to decrease the convergence rate, while large range of search step will reduce the development capability of the bee colony. The parameter *SF* is introduced to control the range of the search step, in order to dynamically adjust the convergence speed and development capability.

The position of bee *i* in the *k*th iteration is memorized as *x_i_*(*k*), the direction of vector (*x_i_*(*k* + 1) − *x_i_*(*k*)) is regarded as the direction of movement for bee *i* in the *k*th iteration, *ϕ* is the angle between the direction of movement for bee *i* and the direction of the best nectar source with greatest value of fitness, *x_best_*(*k*) is the position of the best nectar source found so far after the *k*th iteration. If the angle *ϕ* for bee *i* meets *ϕ* ≤ 90°, the search of bee *i* is successful; else, the search is unsuccessful.

**Figure 3.**
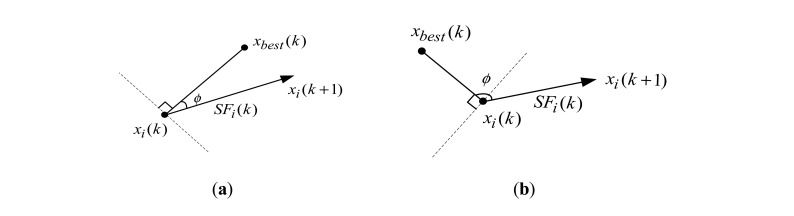
(**a**) Case of enlarging the range of the search step; (**b**) Case of lessening the range of the search step.

According to the definitions above, if the direction of movement for a bee is accordant with the direction of the best nectar source obtained in the current cycle, the size of the search step can be properly increased; otherwise, the range of the search step can be decreased appropriately. The two cases are indicated in Figure 3b.

For both cases of enlarging and lessening the range of the search step, the specific expression of the calculation as defined in Equation (7) depends on experience.

(7) $$SF_{i}(k+1)=\{\begin{array}{ll} SF_{i}(k)\times (1+\cos\phi ) & \;if\;\cos\phi >0\\ SF_{i}(k)/(2-\cos\phi ) & if\;\cos\phi \leq 0 \end{array}$$

The search step should not exceed *SF*_max_ to ensure the stability of the algorithm. Because the search direction is selected randomly in the search process, the size of angle *ϕ* could be described as Equation (8).

(8) $$\cos\phi =\{\begin{array}{ll} \frac{\left(x_{i}(k)-x_{i}(k+1))\cdot (x_{\text{best}}(k)-x_{i}(k)\right)}{\left\|x_{i}(k)-x_{i}(k+1)\right\|\times \left\|x_{\text{best}}(k)-x_{i}(k)\right\|} & if\;rd>0\\ \frac{\left(x_{i}(k+1)-x_{i}(k))\cdot (x_{\text{best}}(k)-x_{i}(k)\right)}{\left\|x_{i}(k+1)-x_{i}(k)\right\|\times \left\|x_{\text{best}}(k)-x_{i}(k)\right\|} & if\;rd\leq 0 \end{array}$$

where ‖ ‖ is the length of two vectors, · is the dot product between two vectors, *rd* is a random number in the range [−1, 1].

Assuming that the range of the search step for bee *i* is *SF_i_*(*k* − 1) = *a*, the search is successful and *ϕ* = 0. According to Equation (7), we have *SF_i_*_+1_(*k*) = 2*a*. If the search in *k*+1th iteration is unsuccessful and *ϕ* = 180°, the best solution is between *x_i_*(*k*) and *x_i_*(*k* + 1) and the individual moves to *x_i_*(*k*) in step of *SF_i_*(*k* + 1) = 2*a*/3 in *k*+1th iteration. That is to say, the search range is always among the interval including the best solution. In the same way, no matter whether the search in *k*+2th iteration is successful or not, the best solution is among the search interval.

#### 3.2.2. Adjusting the Calculation of Transition Probability *P*

In the search process for best solution, the selection pressures are different at different stages of cycles. When the cycle is small, the employed bees with smaller fitness value should have more opportunity to recruit the onlooker bees, in order to maintain a high diversity of the colony; when the cycle is large, it is needed to narrow the range of search to speed up the search process. The problem of adjusting selection pressure is equivalent to the problem of how to calculate the transition probability. The calculation of transition probability in basic ABC algorithm is based on the formula shown in Figure 2, while for the different stages with different selection pressures; the improvements in Equation (9) are conducted to adaptively adjust the calculation of transition probability.

(9) $$P_{i}=e^{-\frac{\beta \cdot c}{\text{MaxCycles}}}\cdot P_{1}+(1-e^{-\frac{\beta \cdot c}{\text{MaxCycles}}})\cdot P_{2}$$

in which

(10) $$\begin{array}{cc} P_{1}=\frac{{\text{fit}}_{i}+{\text{fit}}_{\min}}{\sum_{i=1}^{SN}\left({\text{fit}}_{i}+{\text{fit}}_{\min}\right)}, & P_{2}=\frac{{\text{fit}}_{i}-{\text{fit}}_{\min}}{\sum_{i=1}^{SN}\left({\text{fit}}_{i}-{\text{fit}}_{\min}\right)} \end{array}$$

where *SN* is the number of nectar sources, *fit_i_* is the fitness function value of solution *i*, *fit_min_* is the minimum of fitness function after current cycle, MaxCycles is the maximum of total cycle, *c* is the number of current cycle in the range [1, MaxCycles], *β* is a control parameter.

This probabilistic selection depends on the fitness values of the solutions in the population. From Equation (4), we can know that a fitness value depends on the value of cost function, and the fitness function is a monotone decreasing function. For a minimization problem, the better the food source is, the smaller the value of the cost function will become. The difference for different specific problems is the definition of the cost function.

For the gravity matching algorithm, the gravity information is used as the matching quantity, and the gravity information of the best matching point is expected to converge with the observed gravity information. Therefore, the form of the cost function depends on the absolute value of the difference between the two kinds of gravity information. To simplify the expression, the absolute value of the difference between the gravity information of the best matching point and the observed gravity information can be directly used as the cost function. So, the fitness can be described as:

(11) $$\text{fitness}=1/(1+\left|\Delta g_{\text{obs}}-\Delta g\right|)$$

where Δ*g_obs_* is the observed gravity information, Δ*g* is the gravity information of best matching point.

As described above, *c* changes one by one from 1 to MaxCycles during the searching process. The current search stage can be implied by the proportion of *c* to MaxCycles, which is used to confirm the proportions of *P*_1_ and *P*_2_ to *P_i_* shown in Equation (9). On this basis, the introduction of control parameter *β* aims at controlling the proportions further, and the value of *β* is configurable. Therefore, the adjustment of transition probability depends on the values of *c* and *β*.

*P*_1_ and *P*_2_ shown in Equation (10) are treated as the limit values of *P_i_*, and the expressions of *P*_1_ and *P*_2_ should have the following relation between the expression shown in Equation (12):

(12) $$\{\begin{array}{l} P_{1}\;-{\text{fit}}_{i}/\sum_{i=1}^{SN}{\text{fit}}_{i}={\text{fit}}_{\min}\cdot \frac{SN(\frac{1}{SN}\sum_{i=1}^{SN}{\text{fit}}_{i}-{\text{fit}}_{i})}{\sum_{i=1}^{SN}({\text{fit}}_{i}+{\text{fit}}_{\min})\cdot \sum_{i=1}^{SN}{\text{fit}}_{i}}\;\\ P_{2}\;-{\text{fit}}_{i}/\sum_{i=1}^{SN}{\text{fit}}_{i}={\text{fit}}_{\min}\cdot \frac{-SN(\frac{1}{SN}\sum_{i=1}^{SN}{\text{fit}}_{i}-{\text{fit}}_{i})}{\sum_{i=1}^{SN}({\text{fit}}_{i}-{\text{fit}}_{\min})\cdot \sum_{i=1}^{SN}{\text{fit}}_{i}} \end{array}$$

On the condition that the value of *β* is properly configured, it can be known from Equation (9) that when *c* is small, the value of *P_i_* mainly depends on *P*_1_, that is to say, the employed bees whose fitness values are less than the average will have a greater chance of being selected. When *c* is small, the value of *P_i_* mainly depends on *P*_2_, that is to say, the employed bees whose fitness values are greater than the average will have a greater chance of being selected. The law above can be expressed as Equation (13).

(13) $$\{\begin{array}{lll} P_{2}<{\text{fit}}_{i}/\sum_{i=1}^{SN}{\text{fit}}_{i}<P_{1} & ,if & \text{c is small and}\;{\text{fit}}_{i}\leq \frac{1}{SN}\sum_{i=1}^{SN}{\text{fit}}_{i}\\ P_{1}<{\text{fit}}_{i}/\sum_{i=1}^{SN}{\text{fit}}_{i}<P_{2} & ,if & \text{c is large and}\;{\text{fit}}_{i}>\frac{1}{SN}\sum_{i=1}^{SN}{\text{fit}}_{i} \end{array}$$

**Figure 4.**
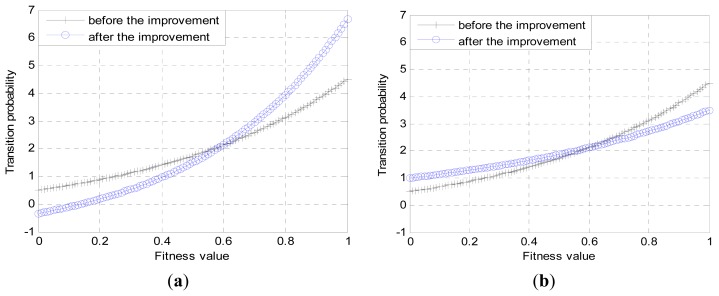
(**a**) Adjustment of transition probability (*c* is small); (**b**) Adjustment of transition probability (*c* is large).

In order to make a clear comparison with the primary selection mechanism, the improved selection mechanism is shown in the form of the curve as in Figure 4a,b.

From Figure 4, we can see that when c is small, the improvement focuses on the promotion of searching precision. When *c* is large, the improvement focuses on the promotion of convergence speed.

## 4. Matching Strategy Based on Improved ABC Algorithm

**Figure 5.**
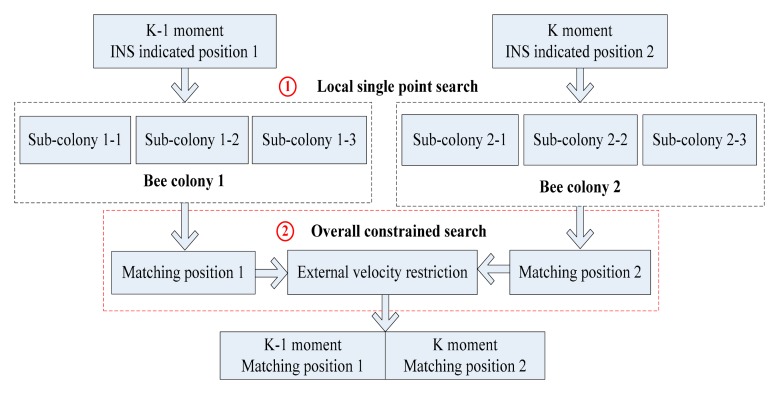
Flow chart of improved ABC search.

Based on the improved ABC algorithm, in order to further enhance the diversity of the colony, the search mechanisms including the main bee colony and sub bee colony are employed, in which the sub bee colony is introduced to assist the main bee colony. Moreover, the gravity matching problem is a complex optimization problem with multiple solutions, and the error of gravity reference database and measurement error of gravimeter cannot be ignored. Even though the search accuracy of the ABC algorithm is high enough, the matching points obtained are also likely to deviate from the true position. To solve this problem, this paper proposes a new search mechanism, using external velocity information to restrain the matching points. Described as Figure 5, both bee colonies search for best matching points in pairs around the adjacent positions indicated by INS with the same search strategy. The local search and overall search are combined organically, in which the local search adopts multiple-group single point search mechanism, and the overall search adopts double-group constrained search mechanism.

### 4.1. Multi-Group Single Point Search (Local)

**Figure 6.**
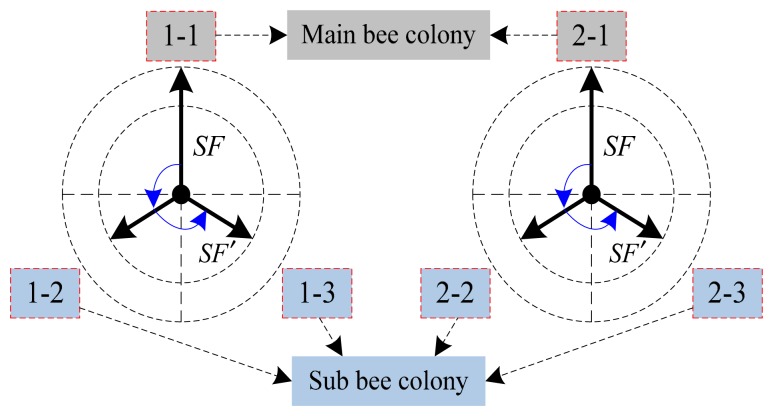
Schematic diagram of multi-group single point search.

As seen in Figure 6, the two synchronous bee colonies (bee colony 1 and bee colony 2) are divided into three groups, respectively. For bee colony 1, the main bee colony 1-1 searches with the improved search mechanism, and the other groups (sub bee colony 1-2 and sub bee colony 1-3) turn 120 degrees in counterclockwise order from the search direction of the main bee colony in sequence. Besides, the range of the search step *SF* is replaced by *SF′* (*SF′* = *rand* (0,1) *SF* < *SF*). After each cycle of search, the individuals in the bee colonies are sorted according to the size of the fitness values. If the nectar source found by an individual from the other two sub bee colonies is significantly better than the nectar source found by an individual in the main colony, the individual in bee colony 1-1 will be replaced.

This local search method with multi-group can increase the diversity of the colony, but time complexity becomes higher. Therefore, the original bee colony will be divided without increasing the size of the bee colony in order to ensure the search accuracy. In detail, the sizes of bee colonies 1-1 and 2-1 are reset to half of the original size, and the numbers of all the other four groups are half of the size of bee colony 1-1 or 2-1.

### 4.2. Double-Group Constrained Search (Overall)

**Figure 7.**
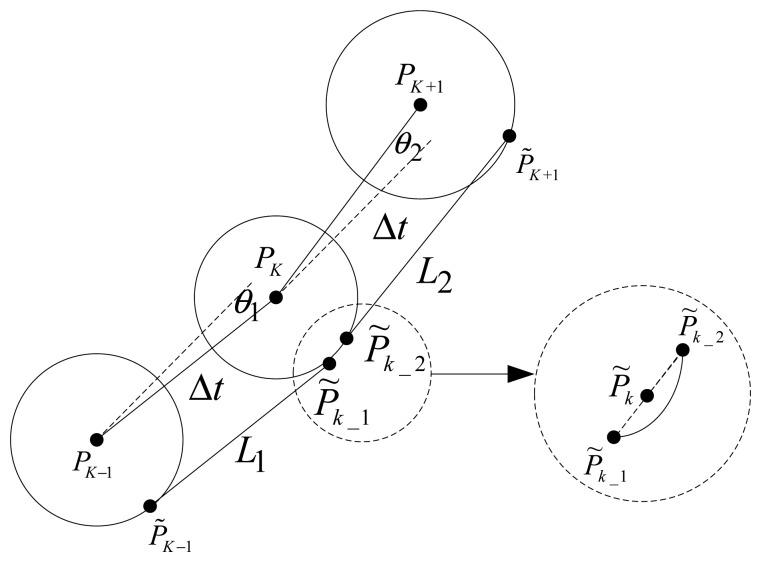
Schematic diagram of double-group constrained search.

Due to the shortcomings of the simple constraint and incidental mismatch in single point search, a double-group constrained search is introduced. As shown in Figure 7, the distance between the two points is restricted with the external velocity information. Even so, more than one possible matching result will be obtained; to solve this problem, the modified Hausdorff distance is used to screen the matching results further.

Shown as Figure 7, *P_k_*_−1_, *P_k_* and *P_k_*_+1_ express the positions of carrier indicated by INS at the moment of *k*−1, *k* and Δ*g_k_ k*+1, respectively. Meanwhile, the gravimeter can output the gravity anomaly values Δ*g_k_*_−1_, and Δ*g_k_*_+1_. The matching results (*P̃_k_*_−1_ and *P̃_k_*) have to meet the following inequalities:

(14) $$\{\begin{array}{l} \left|\left\|{\tilde{P}}_{k-1}-{\tilde{P}}_{k}\right\|-v\cdot T\right|<\sigma_{d}\\ \left|\Delta g_{\text{obs}}(k-1)-\Delta g({\tilde{P}}_{k-1})\right|<\sigma_{g}\\ \left|\Delta g_{\text{obs}}(k)-\Delta g({\tilde{P}}_{k})\right|<\sigma_{g} \end{array}$$

where ‖ *P̃_k_*_−1_ − *P̃_k_* ‖ is the distance between *P̃_k_*_−1_ and *P̃_k_*, *ν* is the external velocity information, *T* is the time interval for adjacent positions indicated by INS, Δ*g_abs_* (*k*) is the gravity anomaly outputted by gravimeter at *k* moment, Δ*g*(*P̃_k_*) is the gravity anomaly of search point *P̃_k_*_−1_ from the gravity reference database (EGM2008) at *k* moment, *σ_d_* and *σ_g_* are the thresholds of distance and difference of gravity anomaly, respectively.

During the search process based on the improved ABC algorithm, the fitness function can be described as Equation (15).

(15) $$\text{fitness}=1/(1+\sqrt{\sum_{i=0}^{1}{\left|\Delta g_{\text{obs}}(k-i)-\Delta g_{}({\tilde{P}}_{k-i})\right|}^{2}})$$

Because of the measured error and reference database error for the gravity anomaly data, the distance threshold and difference threshold of gravity anomalies cannot be too small, in order to make the effort to meet the limits for real position. Though the external constraints are considered, there still is more than one matching result obtained. Therefore, modified Hausdorff distance is introduced to screen the multiple matching results, and modified Hausdorff distance is defined as Equation (16) based on the basic Hausdorff distance [22].

(16) $$d_{MH}(A,B)=\max\{d_{m}(A,B),d_{m}(B,A)\}$$

in which

(17) $$d_{m}(A,B)=\frac{1}{N_{A}}\sum_{a\in A}\underset{b\in B}{\min}\left\|a-b\right\|$$

(18) $$d_{m}(B,A)=\frac{1}{N_{B}}\sum_{b\in B}\underset{a\in A}{\min}\left\|b-a\right\|$$

where ‖·‖ is the distance norm between point-group *A* and point-group *B*, *N_A_* is the number of points in point-group *A*, *N_B_* is the number of points in point-group *B*.

The best position determined as the final result of the matching is needed to satisfy Equation (19).

(19) $$S_{\text{best}}^{}=\left\{S_{i}|d_{MH}(S_{i},S_{\text{obs}})=\min,\;i\leq N\right\}$$

where *N* is number of matching sequence for screening, *S_i_* is the matching sequence *i*, *S_obs_* is the position sequence provided by INS, *S_best_* is the matching sequence output after screening.

It can be seen from the previous matching process that two sets of matching are conducted in every cycle of the matching process, and the average of the two matching results is treated as the final result, similar to “estimates” and “smooth” in the estimation theory.

The specific realization of the search mechanism mentioned above in the gravity matching method is shown as Table 2 in the form of pseudo-code.

## 5. Experimental Analysis

### 5.1. Simulation Experiment Analysis

**Figure 8.**
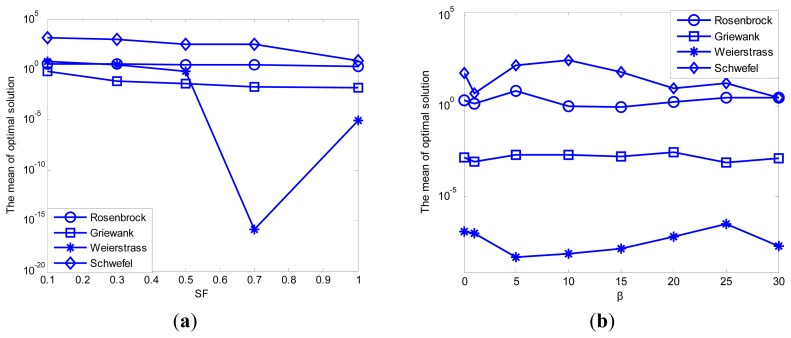
(**a**) Effect of SF on multimodal functions; (**b**) Effect of β on multimodal functions.

Shown as Table 3, four basic test functions (Rosenbrock, Griewank, Weierstrass and Schwefel) are chosen to test the performance of the improved ABC algorithm. The adaptively adjusted range of search step *SF* and adjustment factor *β* for transition probability are the control parameters of the ABC algorithm, and these control parameters are needed to be tuned for better performance. The results presented in Table 4 are demonstrated in Figure 8.

The simulation results shown in Table 4 and Figure 8 mainly focus on the performance analysis of convergence precision and ignore the change in convergence speed. In order to verify the significance of the proposed modification, the following part is added to the simulation experiment analysis.

Because the performance of convergence precision for the test function “Griewank” is worse than the other test functions, the test function “Griewank” is selected to prove the effect of the improvement proposed on performance of convergence speed. The configuration of parameters for ABC algorithm is unchanged. The simulation result can be seen as follows.

**Figure 9.**
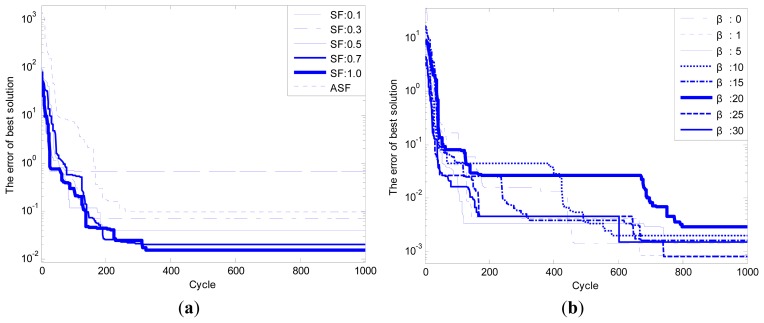
(**a**) Adjustment the search step; (**b**) Adjustment the calculation of transition probability.

Comparing the simulation result shown in Table 4 with the simulation result shown in Figure 9, we can know that for one thing, the advantage of adjusting the search step is not an improvement of convergence precision but convergence speed; for another, the enhancing of convergence precision by adjusting the calculation of transition probability is more obvious than convergence speed. Besides, a comprehensive value of *β* is 10. Therefore, better performance will be achieved if both improvements are introduced to the basic ABC algorithm than a single improvement.

**Figure 10.**
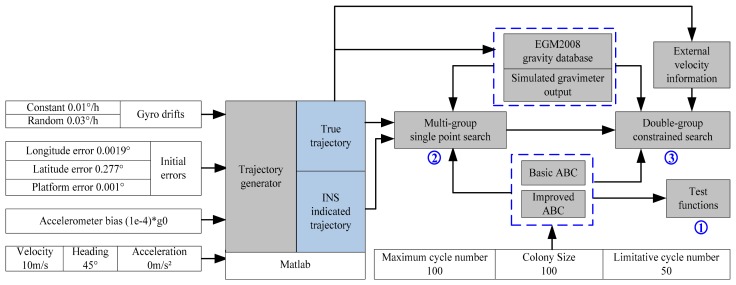
Flowchart of simulation experiment.

The following section outlines the simulation experiment designed for verifying the feasibility of the gravity matching navigation method. In Figure 10, after simulation conditions (INS and ABC algorithm) are determined, the trajectory indicated by INS and the true trajectory are simulated by Matlab, and the matching algorithm is applied. The results of the simulation experiment include:

① The performance comparison between basic ABC algorithm and improved ABC algorithm is made through basic test functions.
② The results of gravity matching navigation based on multi-group single point search are analyzed.
③ The matching results of double-group constrained search using external velocity information are discussed.

**Figure 11.**
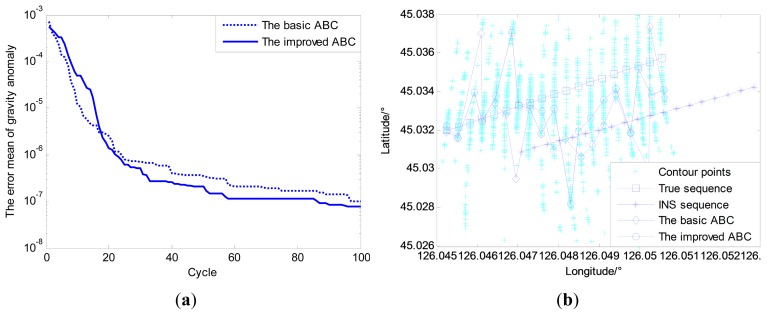
(**a**) Convergence curves for single point search; (**b**) Matching results for single point search.

Under above-mentioned conditions, the improved ABC algorithm is applied to the multi-group single point search. As seen from Figure 11a, the simulation results show that for improved ABC algorithm, the convergence speed and search accuracy are increased by 40.2% and 21.5%, respectively compared with basic ABC algorithm. However, it can be seen from Figure 11b that the matching results of improved ABC algorithm applied to single point matching have no obvious advantages to the basic ABC algorithm due to the contour points.

To test the proposed system clearly, more conditions are tested. The matching points cannot converge to the real points shown in Figure 11. A possible factor causing this result is the poor suitability of the matching area. The gravity information in the matching area greatly influences the matching performance.

**Figure 12.**
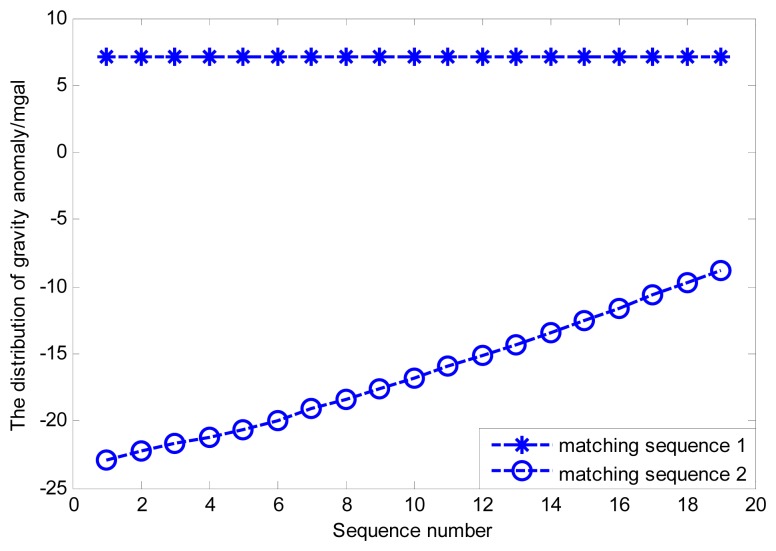
The distribution of the gravity anomaly for different matching sequences.

**Figure 13.**
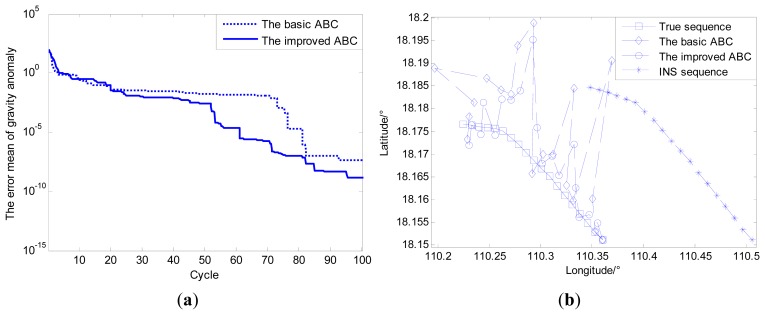
(**a**) Convergence curves for single point search; (**b**) Matching results for single point search.

Therefore, the new simulation area is selected where the change of gravity information is much more obvious than in the old simulation area, and the other simulation conditions remain unchanged. Both the distributions of gravity anomaly for the old and new matching sequences are shown in Figure 12. The simulation results can be seen in Figure 13a,b.

By comparing and analyzing the simulation results shown in Figures 11 and 13, it is easy to find that matching areas with richer gravity information can achieve better performance. The selection of navigation paths with rich gravity information can contribute to the performance of gravity matching. Despite all this, simple improvements in the ABC algorithm involved in the multi-group single point search mechanism cannot solve the problem fundamentally, and the influence of the contour points still exists no matter whether the gravity information of the matching area is rich or poor. Therefore, proper modifications to the search mechanism are needed to enhance the performance of the matching algorithm fundamentally.

**Figure 14.**
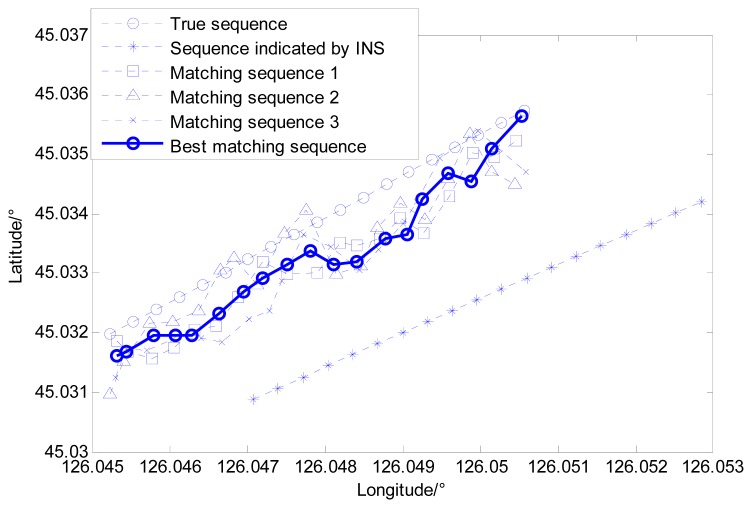
Screening for best matching result by Hausdorff distance.

**Figure 15.**
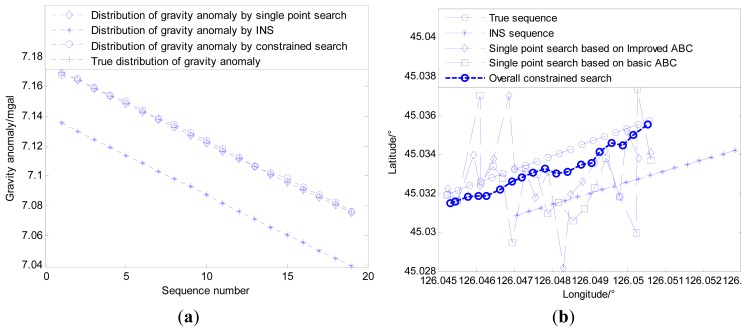
(**a**) Distributions of gravity anomaly; (**b**) Comparison of results for different search strategies.

Based on the simulation result shown as Figure 11, the external velocity information is used to restrain local single point search processes for both bee colonies, and the best matching sequence is screened from the possible matching sequences by the modified Hausdorff distance shown in Figure 14. Figure 15a shows the distribution of gravity anomalies for different matching sequences. In Figure 15b, the matching results with different search strategies are listed for comparison.

It can be concluded from the simulation results that the distribution of the gravity anomaly after multi-group single point matching and the true distribution of the gravity anomaly almost coincide, while the problem of mismatch still exists. Compared with multi-group single point search, the double-group constrained search mechanism can reduce the possibility of mismatch at the cost of some search precision. However, the simulation experiment ignores the measurement error of gravimeter that is inevitable in practice, therefore, it is necessary to conduct further analyses for practical situations.

### 5.2. Ocean Experiment Analysis

**Figure 16.**
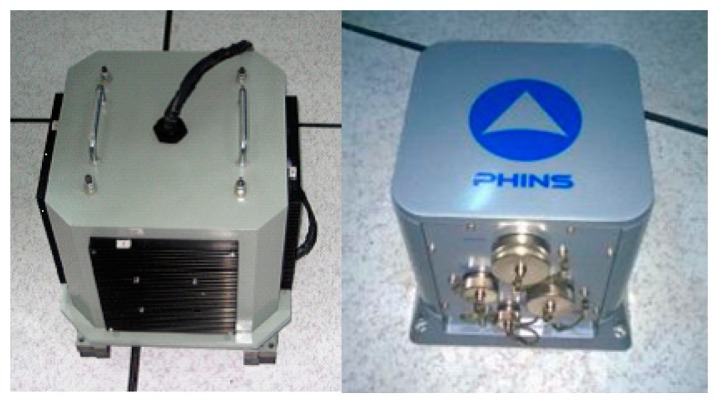
FOG-INS and PHINS.

**Figure 17.**
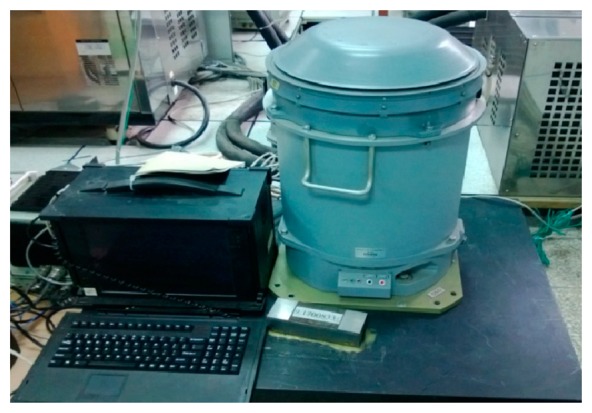
Chekan-AM marine gravimeter.

For further validating the conclusion, the ocean experiment provided with INS and gravimeter was undertaken in this study. FOG-INS and PHINS are two kinds of inertial navigation systems used in ocean experiments shown in Figure 16. FOG-INS is made by our lab and its performance index is described in Table 5, whose positioning error accumulates over time without any revision. PHINS is introduced from IXSEA, the performance index of which is almost the same as FOG-INS, while Table 6 shows that the integrated navigation system composed of GPS and PHINS is used as reference datum for its high navigation precision. Figure 17 shows the Chekan-AM marine gravimeter introduced from Russia, which can provide real-time revised gravity data, and its performance index is shown in Table 7.

**Figure 18.**
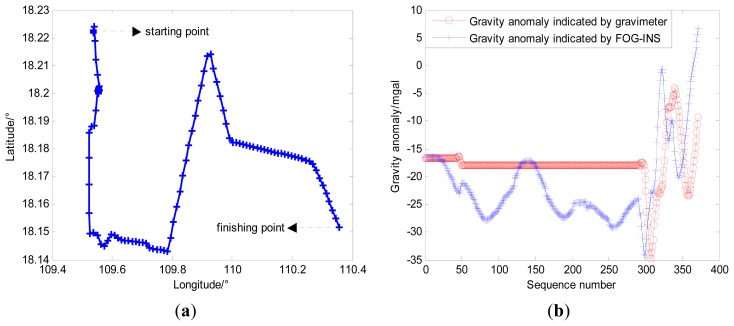
(**a**) True navigation trajectory indicated by PHINS; (**b**) Gravity anomalies from gravimeter and FOG-INS.

**Figure 19.**
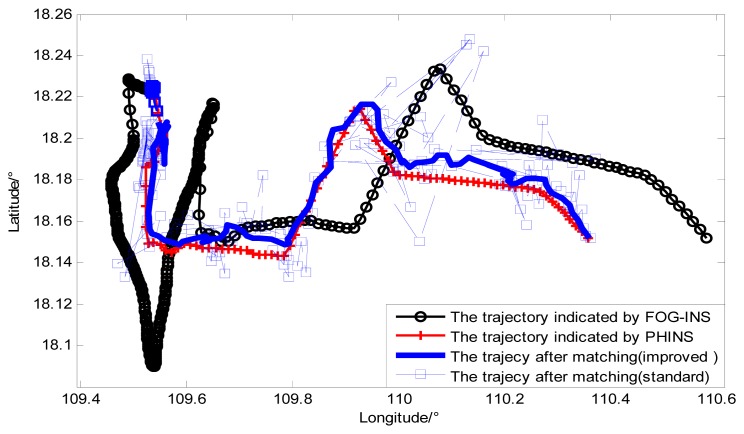
The results of gravity matching in ocean experiment.

In the ocean experiment, both FOG-INS and PHINS provide real-time navigation information of vehicle, and Figure 18a shows the navigation trajectory of the vehicle. Besides, Chekan-AM marine gravimeter provides gravity anomaly information which is synchronous with other navigation information, and the external velocity information is obtained from PHINS. The difference between the gravity anomaly measured by gravimeter and that indicated by FOG-INS can be observed in Figure 18b and the difference of gravity anomaly reflects the position error of FOG-INS indirectly. Figure 19 shows performance comparison between trajectories before matching and trajectory after matching in the ocean experiment, and Table 8 shows the longitude error and the latitude error for trajectories in Figure 19.

From the experiment data in Table 8, we can see that the matching trajectory is closer to the true trajectory compared with the trajectory indicated by FOG-INS, which is consistent with the conclusion from the simulation experiment. The double-group constrained search mechanism based on a multi-group single point search overcomes the influence of contour points and prevents position error from accumulating over time. Besides, it is different from the simulation experiment that the measurement error of the gravimeter was considered in the ocean experiment, which makes the results more reliable.

## 6. Conclusions

The gravity matching navigation problem is a complex optimization problem with multiple solutions. Considering the shortages of state-of-the-art gravity matching algorithms, the gravity matching navigation method with a search strategy based on an improved artificial bee colony algorithm is presented. First of all, better performance of the ABC algorithm is obtained by adaptively adjusting the range of the search step and calculation of transition probability. Secondly, a new search mechanism based on the ABC algorithm is proposed by introducing multi-group single point search and double-group constrained search at the same time. At last, both simulations and experiments are conducted to verify the feasibility of this method. The experimental results show that this method of gravity matching can accurately match the vehicle's position, and effectively decrease the probability of mismatch.

## Figures and Tables

**Table 1.** Relationship between the behavior of bees and the optimization problem.

**Behavior of Bees**	**Optimization Problem**
Position of nectar	Feasible solution
Yield of nectar	Value of fitness function
Honey speed	Converge speed
Greatest gains	Optimal solution

**Table 2.** Improved ABC Algorithm with single point search and constrained search.

1: Start

2: Create randomly an initial population.
3: for *i* = 1 to MaxCycles
4: Produce a new nectar source population *x_i_* for employed bee.
5: Restrict the position of bee colonies; set *x_i_* = *x_max_* or *x_i_* = *x_min_* when *x_i_* > *x_max_* or *x_i_* < *x_min_*.
6: Calculate the fitness function value.
7: Multi-group single point search.
8: Bees in main bee colony are replaced by bees in sub bee colony with higher fitness value.
9: Introduce the external velocity restriction.
10: Calculate the probability values.
11: Two groups of onlooker bees select employed bee by probability.
12: Replace *x_i_* with a new randomly produced solution, if the best solution does not change.
13: end for
14: Screen the matching results using modified Hausdorff distance.
15: Memorize the best solution achieved so far.
16: End

**Table 3.** Basic test functions employed in the simulation experiment.

**Test Function**	**Stagnation Point**	**Search Range**	**Expression**
Rosenbrock	*f*(1⃗) = 0	[−2.048, 2.048]^*D*^	$f(x)=\sum_{i=1}^{D-1}\left[100{\left(x_{i+1}-x_{i}^{2}\right)}^{2}+{\left(x_{i}-1\right)}^{2}\right]$
Griewank	*f*(0⃗) = 0	[−600, 600]^*D*^	$f(x)=\frac{1}{4000}\sum_{i=1}^{D-1}x_{i}^{2}-\prod_{i=1}^{D}\cos(\frac{x_{i}}{\sqrt{i}})+1$
Weierstrass	*f*(0⃗) = 0	[−0.5, 0.5]^*D*^	$$\begin{array}{l} f(x)=\sum_{i=1}^{D}\left(\sum_{k=0}^{k\;\max}\left[a^{k}\cos(2\pi b^{k}(x_{i}+0.5))\right]\right)\\ -D\sum_{k=0}^{k\;\max}\left[a^{k}\cos(2\pi b^{k}0.5)\right],\;a=0.5,\;b=3,\;k\;\max=20 \end{array}$$
Schwefel	*f*(4⃗20.96) = 0	[−500, 500]^*D*^	$f(x)=418.9829xD-\sum_{i=1}^{D-1}-x_{i}\sin(\sqrt{\left|x_{i}\right|})$

**Table 4.** Best results of basic test functions. (Dimension: 2, Colony Size: 10, Run times: 30, Maxcycles: 30,000).

**Test Function**	**1. Rosenbrock**	**2. Griewank**	**3. Weierstrass**	**4. Schwefel**
SF: 0.1	3.89 × 10° ± 1.49 × 10°	6.81 × 10^−1^ ± 8.39 × 10^−1^	6.08 × 10° ± 1.46 × 10°	1.40 × 10^3^ ± 2.61 × 10^2^
0.3	3.79 × 10° ± 1.99 × 10°	7.15 × 10^−2^ ± 5.92 × 10^−2^	2.89 × 10° ± 1.26 × 10°	9.49 × 10^2^ ± 2.19 × 10^2^
0.5	3.22 × 10° ± 2.05 × 10°	3.87 × 10^−2^ ± 2.64 × 10^−2^	6.33 × 10^−1^ ± 7.00 × 10^−1^	3.59 × 10^2^ ± 1.16 × 10^2^
0.7	2.77 × 10° ± 2.26 × 10°	2.00 × 10^−2^ ± 1.59 × 10^−2^	1.18 × 10^−16^ ± 6.38 × 10^−16^	3.20 × 10^2^ ± 1.36 × 10^2^
(basic)1	2.08 × 10° ± 2.44 × 10°	1.57 × 10^−2^ ± 9.06 × 10^−3^	9.01 × 10^−6^ ± 4.61 × 10^−5^	7.91 × 10° ± 2.95 × 10^1^
ASF	6.15 × 10^−1^ ± 6.94 × 10^−1^	9.84 × 10^−2^ ± 1.02 × 10^−1^	1.33 × 10^−8^ ± 4.67 × 10^−8^	1.93 × 10^2^ ± 4.59 × 10^1^

*β*: 0.0	2.17 × 10° ± 4.33 × 10°	1.49 × 10^−3^ ± 3.46 × 10^−3^	1.22 × 10^−7^ ± 1.30 × 10^−7^	6.69 × 10^1^ ± 7.17 × 10^1^
1.0	1.40 × 10° ± 1.56 × 10°	8.48 × 10^−4^ ± 2.53 × 10^−3^	9.25 × 10^−8^ ± 6.21 × 10^−7^	5.16 × 10° ± 8.75 × 10^1^
5.0	6.83 × 10° ± 1.06 × 10°	1.98 × 10^−3^ ± 3.68 × 10^−3^	4.31 × 10^−9^ ± 8.43 × 10^−9^	1.87 × 10^2^ ± 2.76 × 10^2^
10	9.91 × 10^−1^ ± 1.10 × 10^-1^	1.99 × 10^−3^ ± 3.51 × 10^−3^	7.28 × 10^−9^ ± 2.15 × 10^−8^	3.74 × 10^2^ ± 6.42 × 10^1^
15	9.22 × 10^−1^ ± 3.73 × 10^-1^	1.65 × 10^−3^ ± 3.38 × 10^−3^	1.34 × 10^−8^ ± 7.27 × 10^−8^	8.14 × 10^1^ ± 3.89 × 10^1^
20	1.73 × 10° ± 1.66 × 10°	2.97 × 10^−3^ ± 4.00 × 10^−3^	6.35 × 10^−8^ ± 2.26 × 10^−8^	9.63 × 10° ± 5.33 × 10°
25	3.12 × 10° ± 1.61 × 10°	8.07 × 10^−4^ ± 2.26 × 10^−3^	3.29 × 10^−7^ ± 1.52 × 10^−7^	1.91 × 10^1^ ± 2.95 × 10^1^
30	3.01 × 10° ± 1.63 × 10°	1.41 × 10^−3^ ± 3.09 × 10^−3^	1.92 × 10^−8^ ± 4.51 × 10^−7^	3.10 × 10° ± 1.54 × 10°

**Table 5.** The main parameters of inertial components for FOG-INS.

**Parameter Item**	**Fiber Gyro**	**Quartz Accelerometer**
Bias stability	0.01 (°)/h	5 × 10^−5^ g
Random walk	≤0.002 (°)/h	≤1 × 10^−5^ g

**Table 6.** The navigation accuracy of different modes for PHINS.

**Mode**	**Pure Inertial**	**With GPS**
Position	0.6 nm/h	5 ∼ 15 m
Speed	*N/A*	0.1 m/s

**Table 7.** The main parameters of Chekan-AM marine gravimeter.

**Range of Measurement**	**Sensitivity**	**Limits of Permissible Error**	**Drift Rate**
10 *Gal*	0.01 mGal	±1 mGal	3 mGal/day

**Table 8.** Positioning error of gravity matching navigation in the ocean experiment.

**Items**	**Error in Longitude**		**Error in Latitude**	

	**Mean**	**MSE**	**Mean**	**MSE**
Before the matching	3860.5 m	1089.4 m	−389.6 m	34.5 m
After the matching(standard)	621.8 m	264.4 m	−300.6 m	14.4 m
After the matching(improved)	13.7 m	47.6 m	−222.7 m	10.0 m
